# Risk of desmoid formation after laparoscopic *versus* open colectomy and ileorectal anastomosis for familial adenomatous polyposis

**DOI:** 10.1002/bjs5.90

**Published:** 2018-08-08

**Authors:** A. Sinha, E. M. Burns, A. Latchford, S. K. Clark

**Affiliations:** ^1^ The Polyposis Registry, Level 5 St Mark's Hospital Northwick Park, Watford Road, Harrow HA1 3UJ UK

## Abstract

**Background:**

Laparoscopy is used increasingly in prophylactic surgery for patients with familial adenomatous polyposis (FAP) undergoing colectomy with ileorectal anastomosis (IRA). Little is known about the impact of laparoscopy on subsequent desmoid risk. This study documented the risk of desmoid in patients undergoing laparoscopic and open IRA.

**Methods:**

This was an observational study of patients with FAP and known germline *APC* mutation, undergoing IRA at a tertiary referral centre between 1996 and 2016. Patients were retrieved from a prospectively maintained polyposis registry. Data included genotype, family history of desmoid, sex, surgical approach at IRA and postoperative complications. The main outcome was development of either a clinically or radiologically significant desmoid.

**Results:**

Some 112 patients (61 female) underwent colectomy and IRA. A laparoscopic approach was used in 69 patients (61·6 per cent). Baseline characteristics did not differ between patients having an open or laparoscopic approach. Median follow‐up was 5·8 (i.q.r. 2·4–11·2) years. Patients who underwent laparoscopic IRA had a reduced risk of desmoid formation (3 of 69 (4 per cent) *versus* 7 of 43 (16 per cent) in the open group; *P* = 0·043).

**Discussion:**

Laparoscopic IRA may reduce risk of subsequent desmoid formation in patients with FAP.

## Introduction

Familial adenomatous polyposis (FAP) is a dominantly inherited condition resulting from mutation of the adenomatous polyposis coli (*APC*) tumour suppressor gene. It has a population prevalence of between one in 7500 and one in 13 000, with disease penetrance being close to 100 per cent by age 40 years^1^, ^2^. FAP predisposes to the development of hundreds to thousands of adenomatous polyps in the colon and rectum, which inevitably progress to carcinoma if left untreated. These carcinomas occur on average 30 years earlier than sporadic colorectal carcinomas. Patients with FAP are also predisposed to polyposis of the upper gastrointestinal tract, in particular duodenal and periampullary polyposis and desmoid disease^3^. Desmoids are non‐metastasizing, locally invasive myofibroblastic proliferations. The commonest site in patients with FAP is the small bowel mesentery and abdominal wall, although desmoids can occur at virtually any anatomical location. Intra‐abdominal desmoids may cause ureteric or bowel obstruction, and sometimes undergo necrosis. Their presence can make further surgery extremely difficult or even impossible.

Modern surgical management of FAP aims to offer prophylactic surgery, and screening‐detected patients undergo this operation in their late teens or early twenties. As a result of this strategy, desmoid tumours and upper gastrointestinal neoplasia are now the leading causes of mortality in patients with FAP^4^. The choice of surgical prophylaxis in FAP lies between colectomy with an ileorectal anastomosis (IRA) or restorative proctocolectomy (RPC). The practice in the authors' institution is to recommend RPC for patients with a rectal adenoma count exceeding 20, more than 500 colonic adenomas or *APC* mutation between codons 1250 and 1450^5^. IRA is recommended for other patients, as only a minority of these require later proctectomy^6^.

A laparoscopic approach is used increasingly for this prophylactic surgery. Little is known about the impact of laparoscopy and, presumably, reduced surgical trauma on postoperative desmoid development. Less extensive abdominal wounds might result in a reduction in subsequent abdominal wall desmoid formation. This study aimed to establish whether laparoscopic prophylactic surgery is associated with a lower rate of postoperative desmoid formation after IRA.

## Methods

### Patients and setting

This was an observational study of patients with FAP who underwent IRA at St Mark's Hospital, a tertiary referral institute for colorectal pathology in England, between 1996 and 2016. Patients with a known germline *APC* mutation were eligible, and were identified from the Polyposis Registry database. This 20‐year period includes the ‘laparoscopic era’ (2006–2016) and the ‘open era’ (1996–2006). Only patients with follow‐up at this centre were included. Patients with known desmoid tumours at the time of IRA were excluded. During follow‐up, CT was performed only when the patient had complaints or a palpable abdominal mass. Ethical approval was not required for this study as it formed part of service evaluation.

### Data collection

Patient demographics, genotype, family history of desmoid, the surgical approach at IRA and postoperative complications were collected from the Polyposis Registry database and hospital records. These variables appear to be significant in predicting desmoid risk, based on a meta‐analysis of the available literature^7^. A germline *APC* mutation 3′ to codon 1399 was considered predictive of high desmoid risk. Patients who had conversion from laparoscopic to open surgery were considered in the open group.

The main outcome was postoperative development of clinically significant desmoid. This was defined as a symptomatic or radiologically detected intra‐abdominal or abdominal wall desmoid, larger than 4 cm. Any desmoid located within the abdominal wall, intra‐abdominally or at both sites fulfilling these criteria was recorded.

Complications of surgery were graded according to the Clavien–Dindo classification^8^. Grade III (reintervention) and grade IV (life‐threatening) complications were included in a univariable analysis. Those with a lower score were excluded as these more minor complications are unlikely to affect the risk of desmoid development.

### Statistical analysis

IBM SPSS® version 24 (IBM, Armonk, New York, USA) was used to perform all analyses.

Patients were censored at the time of first detection of desmoid or last clinical follow‐up. Fisher's exact test was used to compare family history of desmoid formation, sex, desmoid‐prone mutation, complications and return to theatre in the laparoscopic and open groups, as well as the development of desmoid. Student's *t* test was used to compare age and the Mann–Whitney *U* test was employed to compare duration of follow‐up in the two groups. Kaplan–Meier survival analysis was used to assess the factors associated with desmoid formation, given the difference in duration of follow‐up between the open and laparoscopic groups. Cox regression analysis was used to assess factors correlating with desmoid formation over time. Multivariable analysis was not considered statistically valid as the number of events was too small. *P* < 0·050 was considered to be significant.

## Results

Some 112 patients were included in the study. Median follow‐up was 5·8 (i.q.r. 2·4–11·2) years. Baseline characteristics and perioperative data of the colectomy are shown in *Table* 1.

**Table 1:** Characteristics of patients treated in laparoscopic and open groups

	Laparoscopic (*n* = 69)	Open (*n* = 43)	*P* ‡
Age (years)*	26·8(14·9)	25·3(11·6)	0·576§
Sex ratio (M : F)	32 : 37	19 : 24	0·848
Family history of desmoid	4 (6)	6 (14)	0·179
Desmoid‐prone mutation	5 (7)	4 (9)	0·731
Clavien–Dindo complicationgrade ≥ III			0·731
No	64 (93)	39 (91)	
Yes	5 (7)	4 (9)	
Reoperation	4 (6)	3 (7)	1·000
Duration of follow‐up (months)†	49 (24–83)	199 (140–228)	< 0·001¶

Values in parentheses are percentages unless indicated otherwise; values are

* mean(s.d.) and

† median (i.q.r.).

‡ Fisher's exact test, except

§ Student's *t* test and

¶ Mann–Whitney *U* test.

There were no significant differences between the two groups with respect to perioperative deaths or postoperative complications. *Table* 2 details the complications encountered in the laparoscopic and open groups. *Table* 3 summarizes the findings of the univariable analysis. Family history of desmoid and desmoid‐prone mutation are important risk factors for desmoid tumour development.

**Table 2:** Complications associated with laparoscopic and open surgery

	Laparoscopic (*n* = 69)	Open (*n* = 43)
No complication	57 (83)	38 (88)
Anastomotic leak	3 (4)	2 (5)
Bleeding	4 (6)	1 (2)
Infection	1 (1)	1 (2)
Fistula	1 (1)	1 (2)
Ileus/small bowel obstruction	3 (4)	0 (0)
Pancreatitis	1 (1)	0 (0)

Values in parentheses are percentages.

**Table 3:** Univariable analysis of factors impacting on desmoid formation

	Hazard ratio	*P*
Age (years)	1·00 (0·95, 1·05)	0·918
Sex		
M	1·00 (reference)	
F	3·57 (0·76, 16·81)	0·108
Family history of desmoid		
No	1·00 (reference)	
Yes	8·30 (2·34, 29·43)	0·001
Desmoid‐prone mutation		
No	1·00 (reference)	
Yes	9·24 (2·58, 33·12)	0·001
Clavien–Dindo grade		
< III	1·00 (reference)	
≥ III	0·04 (0·00, 362·13)	0·493
Return to theatre		
No	1·00 (reference)	
Yes	0·04 (0·00, 1453·94)	0·557
Mode of operation		
Open	1·00 (reference)	
Laparoscopy	0·43 (0·11, 1·73)	0·220

Values in parentheses are 95 per cent confidence intervals.

**Figure 1:**
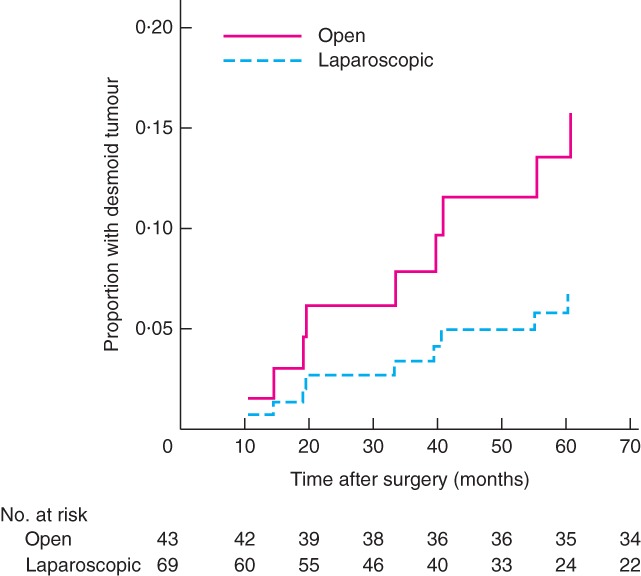
Kaplan–Meier curve showing unadjusted desmoid tumour occurrence after laparoscopic and open colectomy with ileorectal anastomosis

Of ten patients (8·9 per cent) who developed a significant desmoid, the median time from surgery to desmoid development was 3 (i.q.r. 1·5–4·7) years. Four of these ten patients (3 in the laparoscopic group and 7 in the open group) developed an intra‐abdominal desmoid, one developed a desmoid in the abdominal wall, and five developed both intra‐abdominal and abdominal wall desmoids. Neither of two patients who had conversion to an open procedure developed a desmoid. One (1 per cent) of the 69 patients in the laparoscopic cohort developed an abdominal wall desmoid compared with five (12 per cent) of the 43 patients in the open group. After laparoscopic IRA, fewer patients developed desmoid tumours (*P* = 0·043, Fisher's exact test) (*Fig*. 1).

## Discussion

Some 80 per cent of desmoids in patients with FAP have developed by age 35 years, with a median of 3·2 years (range 6 months to 9 years) between prophylactic large bowel surgery and desmoid development^7^, ^9^. This study suggests that laparoscopic IRA for patients with FAP is associated with a lower risk of subsequent desmoid formation compared with open surgery.

The role of previous surgical insult or operative trauma, particularly mesenteric tension, predisposing to desmoid formation is difficult to evaluate. *In vitro* data from cell lines established from desmoid tumour tissue support a role for surgical trauma in desmoid formation. Scratch assays, which can be a surrogate of surgical trauma, carried out on desmoid cell lines show an aberrant cell migration response amongst desmoid cell lines in comparison with control fibroblasts from patients with and without FAP^10^. These data lend credence to the clinical observation of increased desmoid formation following surgical insult. With laparoscopic surgery, the small bowel and its mesentery are not exposed to cooling and drying in the same way as in open surgery, nor to retraction outside the abdomen that might contribute to desmoid development. The associated reduction in desmoid risk is an additional gain for essentially healthy young patients with FAP undergoing this major surgery for prophylaxis.

Analysis of a cohort from the Cleveland Clinic demonstrated that the risk of desmoid development after IRA was less than that of patients undergoing RPC^11^. In the IRA group, a laparoscopic approach at surgery was associated with a lower risk of desmoid development than an open approach. Conversely, in the RPC group, a laparoscopic approach was associated with a higher risk of desmoid development compared with an open approach. RPC was not investigated in the present study.

There was no routine screening for desmoids in this study. Therefore, only larger desmoids and those with clinical impact, rather than all desmoids, could be detected. It is, nevertheless, these large and symptomatic desmoids that cause mortality and morbidity, and are thus of greater clinical importance than small desmoids detected incidentally on imaging.

The main limitation of this study relates to its observational nature and the shorter median follow‐up of patients in the laparoscopic group along with the relatively small sample size. Although follow‐up was shorter, the majority of desmoids will have been detected as 80 per cent of patients with FAP who develop postoperative desmoid generally develop these within 15–18 months of surgery^7^. The relatively small number of confirmed desmoids does not allow conclusions to be drawn with respect to differences between formation rates of abdominal wall and intra‐abdominal desmoids. In addition, the study, despite reporting one of the largest cohorts in the literature, is underpowered to detect independent risk factors.

## Disclosure

The authors declare no conflict of interest.
